# Feasibility of reusing online-generated treatment plans for adaptive radiotherapy in prostate cancer

**DOI:** 10.1016/j.phro.2025.100892

**Published:** 2025-12-13

**Authors:** Sarah A. Mason, Bethany Williams, Sophie Alexander, Alex Dunlop, Alison Tree, Emma J. Harris, Helen McNair

**Affiliations:** a Division of Radiotherapy and Imaging, Institute of Cancer Research, London, UK; b Radiotherapy Department, Royal Marsden NHS Foundation Trust, Sutton, UK

**Keywords:** Online adaptive radiotherapy, Radiotherapy workflow, Prostate cancer radiotherapy, MR-linac

## Abstract

**Background and Purpose:**

: Online adaptive radiotherapy (oART) is underused as generating a treatment plan at every fraction is slow and resource intensive. One method to address this involves reusing plans generated online in previous fractions with similar anatomy. However, manually assessing the suitability of each pre-existing treatment plan is prohibitively time-consuming. To gauge potential impact and motivate the development of software to enable plan recycling, we assessed a strategy whereby *all* pre-existing plans were considered for subsequent fractions in nine hypofractionated prostate patients treated on the magnetic resonance (MR) linear accelerator.

**Methods:**

The verification MR was used to estimate the delivered dose after adaptation to establish a Current Clinical Practice Benchmark. Each structure from the daily MR was propagated backwards onto the reference and daily MRs from previous fractions to calculate the dose to each structure that would have been received had the corresponding plan been delivered. The resulting dose statistics were assessed against: (A) standard target and organ-at-risk objectives, (B) the Current Clinical Practice Benchmark, and (C) circumstances where a pre-existing plan would have matched or outperformed the online plan.

**Results:**

The median [interquartile range] percentage of fractions with at least one acceptable pre-existing plan was 25% [20%], 40% [35%], and 60% [20%] for criteria A, B, and C respectively. Reusing the reference plan was only acceptable in 0%–20% of fractions.

**Conclusion:**

Reusing pre-existing plans is feasible and could accelerate oART and reduce hospital resources in approximately 40% of fractions whilst achieving the same dose-volume metrics as current oART workflows.

## Highlights

• Treatment plan repositories enable efficient treatment adaptation.
• Up to 60% of fractions could be treated with reused treatment plans.
• Standard reference treatment plan alone acceptable in ≤20% of fractions.

## Graphical abstract

**Figure:**
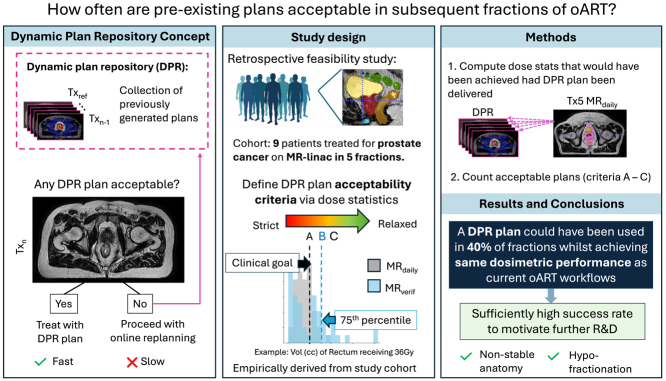

## 1 Introduction

Online adaptive radiotherapy (oART) provides unprecedented opportunities to deliver highly conformal treatment plans that ensure target coverage and minimize dose to healthy tissues even in the presence of large anatomical motion and deformation [1]. Although many oART platforms are available [2], [3], [4], [5], the basic premise of using oART to manage interfraction motion is the same: a daily image is acquired after the patient is set-up for the purpose of generating a radiotherapy plan optimized to the internal anatomy at that time and delivered *in situ*.

Although oART can provide significant improvements in dose distribution [6], [7], [8], [9], few patients (approximately six percent in high and middle income countries [10]) receive this treatment. Reasons for this include high resource burden and the long treatment times [10]. For example, the average oART prostate treatment on an magnetic resonance scanner combined with a linear accelerator (MR-linac) was found to be 45 min [11], compared with 10-15 min for non-adaptive treatments. The two main bottlenecks in oART are contour generation [12], [13], [14] and plan optimization/checking [2]. The long treatment times limit patient access and may degrade treatment quality due to intrafraction motion between image acquisition and irradiation.

Full online replanning may not be necessary in every fraction, especially where there is little interfraction motion [15], [16], [17], [18]. De Leon et al. implemented a plan-of-the-day-like protocol using a library comprised of the reference plan and online plans generated in the first week of treatment (up to fraction five) from for use in all subsequent fractions [16]. This approach was exclusively used in sites with stable anatomy such as oligometastatic lymph nodes, and was thus only implemented in 5.6% of oART treatments. In a retrospective MR-linac study of five-fraction pancreatic radiotherapy, Nasief et al. found the reference plan acceptable in 28% of fractions and developed a machine learning classifier that identified such fractions with 98% accuracy [15]. Similarly, in 25-fraction cervical cancer radiotherapy, Sun et al. found that 27% of fractions would have achieved clinically acceptable dose statistics with the reference plan, and developed a deep learning model using daily and reference images and contours to identify such cases with 91.7% accuracy [19].

We expanded on these concepts of reusing a single or small subset of reference plans to posit that an unlimited number of pre-existing plans (a Dynamic Plan Repository (DPR)) could be treatment candidates. Unlike previous library-based oART approaches that assume a site-specific schedule of replanning regardless of patient-specific anatomical changes or only consider a limited number of reference plans, the DPR concept could enable daily ad-hoc decisions as to whether or not to replan based on consideration of *every* available plan. The aim of this study was to determine how often a DPR plan would be acceptable, using data from oART treatments for prostate cancer as a test case.

## 2 Materials and methods

### 2.1 Clinical radiotherapy protocol

Data from nine patients who received hypofractionated radiotherapy (36.25 Gy in five fractions [20]) for prostate cancer on the Elekta Unity MR-linac (Elekta AB, Stockholm, Sweden) were used. These patients were treated under the Prospective Evaluation of Radiotherapy Using Magnetic Resonance Image Guided Treatment (PERMIT; NCT03727698) and Multiple Outcome Evaluation of Radiotherapy Using the MR-linac (MOMENTUM NCT04075305) studies [21], [22] and consented for images to be used in research.

At every fraction, an online ‘adapt to shape’ (ATS) workflow [2] was adopted, during which two T2-weighted 3D MR images (MR_daily_ and verification MR [MR_verif_]) were acquired and imported into online Monaco (Monaco TPS, Elekta AB, Sweden V5.40.01). Contours for the prostate, seminal vesicles, and all OARs except for bladder and urethra were initialized by deformably propagating the reference structure set to the MR_daily_ using Monaco. The bladder and urethra were initialized by rigidly propagating the reference structures set to the MR_daily_. All propagated structures within 2 cm of the planning target volume (PTV) were then manually corrected to accurately represent the anatomy. Margin expansions (Supplementary A, Table S1) were used to recreate clinical target volumes (CTVs) including the prostate and prescribed length of seminal vesicles (CTV_psv_ and CTV_sv_) from which PTVs were generated. An online plan was optimized using MR_daily_ image contours and the reference plan parameters. The reference plan used (PLAN_ref_) was the offline-generated computed tomography (CT) reference plan (CT_ref_) for fraction 1 (Tx1) and the online-generated plan at Tx1 (MR_ref_) for all subsequent fractions. The MR_verif_ was used to check target coverage of the plan generated online. If target coverage was acceptable, the plan was approved and delivered. Otherwise, an ‘adapt to position’ (ATP) of ATS workflow was applied [11], [23] to virtually shift the couch to ensure coverage of the prostate by the PTV of the online plan. Specifically, the virtual couch shift was performed by applying Segment Aperture Morphing followed by segment weight optimization [2]. If further improvements were needed, Segment Aperture Morphing followed by segment weight and shape optimization was performed [2].

### 2.2 Offline contour generation

As contours outside of the PTV + 2 cm were unedited online and often erroneous (Supplementary B), they could not be used to accurately calculate dose statistics for the MR_daily_. The MR_verif_ was not contoured online. Refined contours were retrospectively generated offline for both MR_daily_ and MR_verif_ images and used for all dose-volume metric analyses in this work. High contouring accuracy in this context has been previously demonstrated [13], [14].

### 2.3 Calculation of the Current Clinical Practice Benchmark

**Fig. 1:**
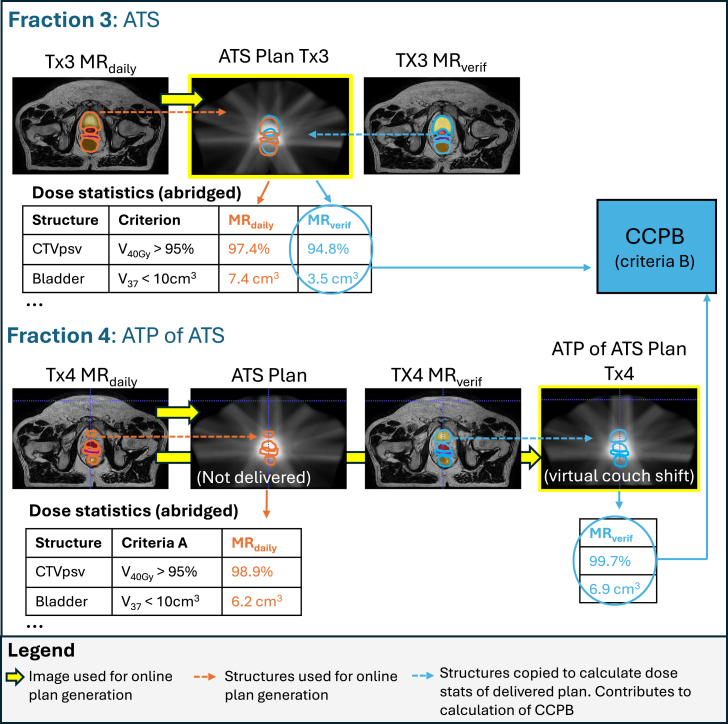
Depiction of MR_verif_ contour propagation to the clinical plan (yellow box) for ATS and ATP of ATS workflows (exemplified by Fraction 3 and Fraction 4 of Patient 1). The doses to these structures for every patient (see abridged dose statistics tables) were pooled into the Current Clinical Practice Benchmark (CCPB) for deriving Acceptability Criteria B.

The MR_verif_ better represents the anatomy during dose delivery than the MR_daily_, and was thus used for Current Clinical Practice Benchmark (CCPB) estimation. MR_verif_ contours were rigidly copied to the dose distribution derived from the approved clinical plan defined on the MR_daily_ to extract the dose statistics for each relevant clinical goal (Fig. 1). Monaco automatically accounted for the density overrides of the copied contours to provide an updated calculation of dose-volume metrics based on MR_verif_ anatomy. In ATP of ATS workflows, the virtual couch shift was performed using Segment Aperture Morphing followed by a weight optimization (and an additional weight and shape optimization, if necessary) on the segments from the clinically approved plan [2].

### 2.4 Dynamic Plan Repository assessment

**Fig. 2:**
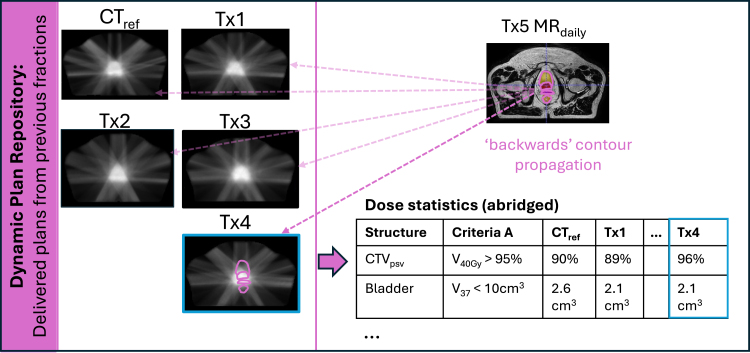
Representation of how the contours on the MR_daily_ from Tx5 were propagated backwards to the Dynamic Plan Repository (DPR) plans to compute the dose statistics that would have been achieved for each plan. The table is an abridged example of the dose statistics that would have been achieved had the DPR plans been delivered at Tx5.

At fraction n+1 the DPR comprised the CT_ref_ (DPR_0_) and all adaptive plans generated as part of the oART workflow up to fraction n (DPR_0, 1, …n_). After MR_daily n+1_ to MR_DPR 0, 1, …n_ registrations, the MR_daily n + 1_ contours were rigidly copied “backwards” to each DPR plan to estimate the dose statistics that would have been achieved had those plans been delivered (Fig. 2). The reason for propagating contours from image to dose (rather than recalculating the dose given a new contoured image) was to alleviate time and resource constraints that would have otherwise prohibited our analyses.

For example, at Tx1, only the CT_ref_ (DPR_0_) was available, so one structure set propagation was performed. At Tx5, there were five plans in the DPR (CT_ref_ and the oART plans generated from Tx1-Tx4), giving 5 structure set propagations. A five-fraction prostate treatment gives a total of fifteen MR_daily_ structure set propagations. For Patient 5, the data from fraction five was corrupted and unusable. A total of 44 fractions from nine patients (130 propagations) were assessed.

MR_daily_ contours were used instead of MR_verif_ contours to evaluate DPR plans because a MR_verif_ would not exist for cases where the DPR plan was acceptable. In such instances, treatment would proceed directly using the DPR plan, analogous to a conventional non-adaptive workflow in which time-consuming contouring and plan optimization are unnecessary, eliminating the need for verification imaging.

### 2.5 Defining plan acceptability criteria

To help standardize decision-making on the MR-linac, we employed online dose objectives for five-fraction prostate cancer treatments for defining plan acceptability in clinician-independent workflows (Table 1). The dose delivered to each individual structure was characterized as either optimal, mandatory, marginal, or unacceptable. Optimal was defined as the ideal value, but violating this constraint did not make a plan unacceptable. Mandatory thresholds *must* be achieved for all OARs (all fractions) and targets (at least four out of five fractions). Marginal acceptability was when slightly lower target coverage was permitted for one fraction only. We adopted these individual structure constraints as the starting point for defining three different acceptability criteria:

**Criteria A - Clinical goals:** Every structure met the mandatory dose constraints outlined in Table 1. Marginal target coverage was only allowed for one fraction. These criteria were strict and are not always met in clinical practice.

**Criteria B - Current clinical practice benchmark (CCPB):** Criteria B was empirically derived from the CCPB (Section 2.3). New thresholds based on the distribution of a specified metric obtained from each structure listed in Table 1 for all available patients were considered. Specifically, for target structures, the 25th percentile metric values were compared with the thresholds given in Table 1; the lower (more permissive) value was set as the new threshold. Likewise, for OAR structures, the 75th percentile was compared with Table 1 thresholds; the greater value was set as the new threshold.

**Criteria C - Exceptional circumstances in current clinical practice:** Criteria C replicate Criteria B with allowable violations in dose constraints in exceptional circumstances (e.g. extreme intrafractional motion) which were assessed on a per-fraction basis. Specifically, if the dose constraints outlined in Criteria B were violated by any structure in the approved clinical plan (according to the MR_verif_) the DPR plans were inspected. If a DPR plan provided equivalent or better dose statistics than the approved clinical plan, it was considered acceptable.

A plan that was unacceptable according to the criteria defined above did not necessarily indicate clinical unacceptability and vice versa.

The median and interquartile range (IQR) frequency of DPR plan acceptability were calculated for each set of criteria. The median and IQR frequency of reference plan acceptability (CT_ref_ and PLAN_ref_) were also reported.

**Table 1:** Normal text: Online dose constraints for 5-fraction prostate cancer treatments for clinician-independent workflows used to define Criteria A. **Bold text**: Dose constraints updated based on empirically-derived data for Criteria B (if more permissive than Criteria A). Note, unchanged dose constraints for Criteria B are also in normal text.

Structure criterion	Optimal	Mandatory		Marginal	Unacceptable	
		A	**B**		A	**B**
CTVpsv V_40Gy_ [%]	95%	90%	90%	85%	<85%	<85%
PTVpsv V_36.25Gy_ [%]	95%	90%	90%	85%	<85%	<85%
PTVpsv D_98%_ [Gy]	34.4 Gy	33.71 Gy	**32.38 Gy**	–	<33.71 Gy	<**32.38 Gy**
Bladder V_37Gy_ [cm^3^]	5 cm^3^	10 cm^3^	10 cm^3^	–	>10 cm^3^	>10 cm^3^
Bladder V_18.1Gy_ [%]	–	40%	40%	–	>40%	>40%
Rectum V_36Gy_ [cm^3^]	1 cm^3^	2 cm^3^	**3.04 cm^3^**	–	>2 cm^3^	>**3.04 cm^3^**
Rectum V_29Gy_ [%]	–	20%	20%	–	>20%	>20%
Rectum V_18.1Gy_ [%]	–	50%	50%	–	>50%	>50%
Bowel V_30Gy_ [cm^3^]	–	1 cm^3^	1 cm^3^	–	>1 cm^3^	>1 cm^3^
Bowel V_18.1Gy_ [cm^3^]	–	5 cm^3^	5 cm^3^	–	>5 cm^3^	>5 cm^3^
Urethra V_42Gy_ [%]	50%	100%	100%	–	–	–

**Table 2:** Number (*N*) and percent of fractions where a DPR plan was considered acceptable according to Criteria A, B, and C for each patient (n = 9). IQR = interquartile range, SD = standard deviation.

Patient	Criteria A		Criteria B		Criteria C		Fractions
	N	%	N	%	N	%	N
P1	2	40	3	60	3	60	5
P2	1	20	3	60	3	60	5
P3	0	0	0	0	2	40	5
P4	2	40	2	40	2	40	5
P5	1	25	1	25	1	25	4
P6	1	20	3	60	3	60	5
P7	3	60	3	60	4	80	5
P8	2	40	3	60	3	60	5
P9	1	20	1	20	1	20	5

**Median** [IQR]	**1** [1]	**25** [20]	**2** [2]	**40** [35]	**3** [1]	**60** [20]	**5** [0]
**Mean** [SD]	**1.44** [0.88]	**29** [17]	**2.00** [1.12]	**41** [22]	**2.44** [1.01]	**49** [19]	**4.89** [0.33]

## 3 Results

### 3.1 Estimation of current clinical benchmark

**Fig. 3:**
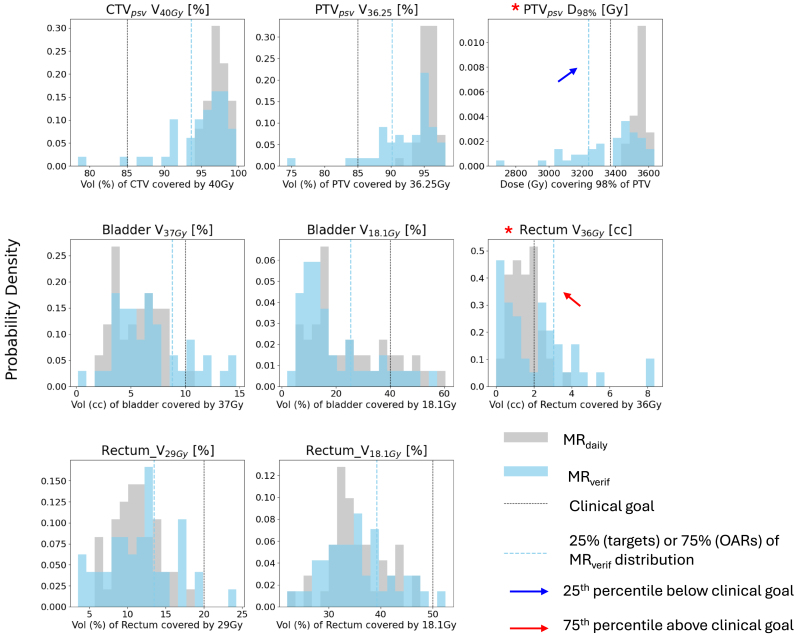
Distribution of dose metrics when the dose from the approved clinical plan was calculated on MR_daily_ (gray) and MR_verif_ (blue) [n = 44 images from 9 patients]. Black dotted lines indicate the lowest/highest acceptable threshold for each structure (either Mandatory or Marginal, if available) as specified in Criteria A. The 25th percentile and 75th percentile of the verification dose metric distribution is indicated by the dotted blue lines for the target and OAR structures, respectively. Structures where the IQR fell outside of the thresholds specified by Criteria A are denoted by a red asterisk, with the IQR boundary indicated with a blue or red arrows for underdosage and overdosage respectively. Corrections for intfrafraction motion in cases where an ATP of ATS workflow was followed were accounted for (i.e. the dose was calculated after the virtual couch shift), so instances of low MR_daily_ target coverage represented here are genuine.

Fig. 3 demonstrates the distribution of dose metrics considered. There was a skew towards poorer target coverage and increased rectal dose when the approved clinical plan was recalculated on the MR_verif_. The 75% or 25% of the dose-volume metric criteria on the MR_verif_ fell outside of the clinical constraints in 2 cases: (1) the dose covering 98% of the PTV, and (2) the volume of rectum covered by 36 Gy, and were thus used to update Criteria B as shown in Table 1.

### 3.2 Evaluation of plan acceptability

The median [IQR] percentage of fractions that had at least one acceptable plan in the DPR was 25% [20%], 40% [35%], and 60% [20%] for criteria A, B, and C, respectively (Table 2). The rate of DPR plan acceptability varied between patients: there were no acceptable DPR plans for all five fractions for patient P3, while acceptable doses would have been delivered in 20% to 80% of fractions using a DPR plan for all other patients assessed. In comparison, CT_ref_ and PLAN_ref_ plans alone were rarely acceptable, with medians of 0% and 20% of fractions respectively meeting Criteria B constraints (Supplementary C, Tables S4 and S5, respectively).

**Fig. 4:**
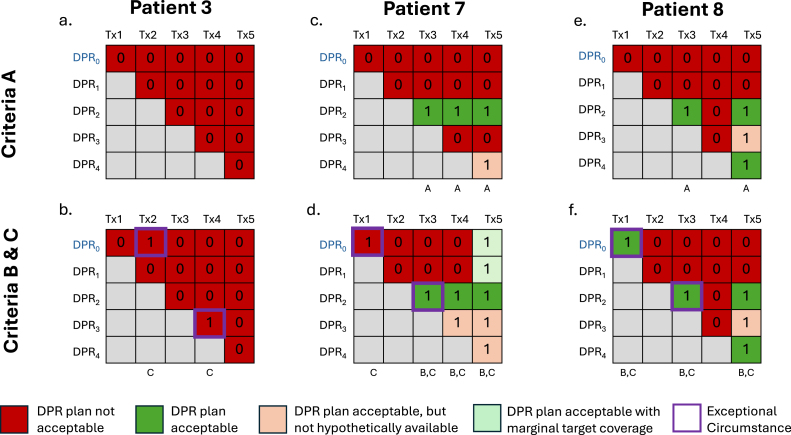
Case studies of 3 patients demonstrating a range of outcomes in DPR plan acceptability. The heatmaps in the top row are colored to indicate which plans in the DPR were acceptable according to Criteria A for all available fractions. The heatmaps in the bottom row are colored to indicate which plans in the DPR were acceptable according to Criteria B, with specific cells outlined in purple to indicate an exceptional circumstance according to Criteria C (i.e. when a plan in the DPR would have matched or improved upon the approved clinical plan based on the dose stats calculated on MR_verif_). An ‘A’, ‘B’, or ‘C’ displayed in small text under each Tx column indicates DPR plan acceptability for that fraction; summing these yields the total number of fractions where a plan from the DPR would have been acceptable according to that criteria. Note: Criteria C is an extension of Criteria B, so any plan acceptable according to Criteria B will also be acceptable according to Criteria C.

Fig. 4 depicts case studies of three patients in this dataset to demonstrate the inter-patient variability in DPR plan acceptability. Patient 7 exemplifies every scenario considered (including exceptional circumstances) and is described in detail in Supplementary D to aid in the interpretation of Fig. 4.

## 4 Discussion

In this pilot study, a substantial proportion of fractions (25%–60% depending on how acceptability is defined) could have been fast-tracked by using a DPR plan for five-fraction prostate cancer patients. There was at least a three-fold and two-fold increase in the frequency of DPR plan acceptability compared with CT_ref_ and PLAN_ref_, respectively, highlighting the benefit of having a repository of plans rather than a single reference. This suggests an exciting possibility of using an ‘adapt to plan’ type adaptation strategy for eligible fractions whilst still achieving ‘adapt to shape’ type dose statistics. These results are sufficiently promising to motivate investigation in larger patient cohorts to confirm potential clinical impact.

There is drive to speed up existing oART workflows, with extensive efforts focused on improving image quality [24], [25], [26], [27], automatic contour generation [28], [29], [30], [31], contour deformation [32], and fast plan reoptimization [33], [34]. These concepts have been incorporated into commercial oART delivery systems to some extent [2], [3], [35], [36] making the process faster than conventional off-line treatment planning. However, the timeline of widespread availability and clinical adoption of emerging technologies to further accelerate online replanning is unclear, underscoring the importance of developing alternative strategies for improving oART efficiency.

There were several limitations to this work and the DPR concept. Although using dose constraints as our only criteria for determining plan acceptability provides a quantitative objective means of analysis, this is an oversimplification of the decision-making process, and that other factors such as dose distributions, clinical outcomes, and patient-specific circumstances are considered in practice. The dose constraints empirically derived for the DPR Current Clinical Practice Benchmark were used to give a better sense of how DPR plans compare with online plans generated in the current workflow. There were also cases in which a DPR plan was never acceptable (see Patient 3 in Fig. 4). In this case, this patient’s prostate swelled over the course of treatment such that adequate target coverage was not possible using DPR plans. Additionally, the DPR concept may have limited benefit in cases where margins or the number of fractions is reduced even further, such as two-fraction prostate cancer radiotherapy treatments. We have initially proposed a naive workflow where an acceptable DPR plan should be delivered at every opportunity, but appreciate that there may be alternative workflows (e.g. always performing full online replanning at fractions one and two given how infrequently the CT_ref_ and PLAN_ref_ were acceptable) that could increase the probability of successful DPR usage in later fractions.

Although the prostate is the most commonly treated site with oART [37] and is widely relevant and has sufficient data to support the potential impact of the DPR concept, other anatomical sites may reap greater benefits. For example, Sun et al. demonstrated that 179/671 fractions from a cohort of 24 patients receiving radiotherapy for cervical cancer could have been delivered using a single reference plan rather than requiring online replanning [19]. Cervical cancer treatment is typically delivered over 25 fractions [38], and because the DPR contains *all* online-generated plans (not just those from the first three to five fractions), the likelihood of reusing a pre-existing plan could increase over the course of treatment. Incorporating DPR plans as treatment candidates could therefore further expand this proportion and amplify the overall clinical impact.

Based on these promising results, we will develop the software tools required to identify whether DPR plans are acceptable, prioritizing techniques that are *site-independent* to ensure the DPR concept could be translated to areas with the greatest clinical need. Considerations for software development include: fast processing time given that a potentially large number of plans need to be assessed, a plan ranking system or probabilistic output to handle cases where multiple DPR plans are considered acceptable (exemplified by Patient 7, Tx5 in Fig. 4) and the inclusion of dose in addition to imaging data. The latter is desired because the allowable differences between image pairs will vary depending on the dose gradients employed, and would thus be dependent on the fractionation schedule and anatomical site. Expanding on the work by Nasief et al. and Sun et al. we are currently developing AI-based solutions designed to run in the background immediately after MR_daily_ acquisition to quickly identify any acceptable DPR plans without the need for manual intervention/segmentation to ensure clinical feasibility [15], [19]. Deployment of this software prototype in future offline observer studies will allow further investigation of usage patterns to guide more efficient resource allocation (e.g. staffing and scheduling logistics) to maximize the DPR benefits.

The DPR concept is not meant to *replace* online replanning, as it is well established that adaptation is often needed to account for interfractional motion [39], [40]. Rather, it is a tool that is meant to opportunistically leverage the multitude of plans generated as *part of* online replanning to streamline the process when possible. In the scenario in which time and staffing requirements between full online replanning and treating with an acceptable DPR plan are equivalent, then the question of whether or not to replan is subject to debate. In the meantime, the DPR concept could be an elegant, vendor-agnostic way to bypass the bottlenecks and resource requirements of oART while still providing equivalent benefit in terms of dose-volume metrics for eligible fractions. Furthermore, the software tools to support DPR implementation would ultimately assess plan acceptability, which could also have roles in quality assurance, and identifying the most appropriate plan to use as a starting template to facilitate faster optimization and simulation-free planning [41], [42], [43].

In conclusion, this work suggests that online plans could be more effectively reused in treatments with non-stable anatomy and short fractionation schedules. Adaptive radiotherapy does not necessarily require full online replanning *every day* to be advantageous. The DPR could provide appreciable clinical benefit by fast-tracking oART, even with under strict acceptability criteria. Our results motivate further developments in plan selection tools needed to bring this concept to fruition.

## CRediT authorship contribution statement

**Sarah A. Mason:** Conceptualization, Methodology, Software, Validation, Formal analysis, Writing – original draft, Visualization. **Bethany Williams:** Investigation, Data curation, Writing – review & editing. **Sophie Alexander:** Investigation, Data curation, Writing – review & editing. **Alex Dunlop:** Methodology, Validation, Writing – review & editing. **Alison Tree:** Writing – review & editing, Supervision, Funding acquisition. **Emma J. Harris:** Conceptualization, Methodology, Writing – review & editing, Supervision, Project administration. **Helen McNair:** Conceptualization, Writing – review & editing, Supervision, Project administration, Funding acquisition.

## Declaration of competing interest

The authors declare the following financial interests/personal relationships which may be considered as potential competing interests: All authors acknowledge NHS funding to the NIHR Biomedical Research Centre at The Royal Marsden and The Institute of Cancer Research, London. Professor McNair, Bethany Williams, and Sarah Mason are funded by The Royal Marsden Cancer Charity and supported by the National Institute for Health Research (NIHR) Biomedical Research Centre at The Royal Marsden NHS Foundation Trust and the Institute of Cancer Research, London (ICA-SCL-2018-04-ST2-002). Sarah Mason also received salary support from Elekta for an unrelated project. The Royal Marsden NHS Foundation Trust and The Institute of Cancer Research receive research funding from Elekta as part of the MR linac consortium. Dr Tree is supported by the Cancer Research UK Radiation Research Centre of Excellence at The Institute of Cancer Research and The Royal Marsden NHS Foundation Trust (grant refs: A28724 and RRCOER-Jun24/100006) and a Cancer Research UK Programme Grant (ref: C33589/A28284). Dr Tree also received honoraria from Elekta, Astellas, and Bayer. Dr Harris is supported by a Cancer Research UK Programme Foundation Award (C20892/A23557).

## Appendix A Supplementary data

MMC S1 Documents, tables, and images for supporting and expanding upon study methods and results. MMC S1
