# Supplementary material for: Feasibility of reusing online-generated treatment plans for adaptive radiotherapy in prostate cancer
Source: Phys Imaging Radiat Oncol. 2025 Dec 13;37:100892. doi: 10.1016/j.phro.2025.100892 (PMC12771498; doi:10.1016/j.phro.2025.100892)
Supplement: MMC S1 — Documents, tables, and images for supporting and expanding upon study methods and results. [file mmc1.pdf]

Supplementary A: Pre-treatment imaging and planning protocol

As part of standard protocol treatment preparation, patients were (1) asked to empty their bladder 30 minutes prior to all imaging and treatment procedures and then drink 350 mL of water and (2) undergo enemas 2 days prior to treatment and within 2 hours of each fraction. Before radiotherapy, computed tomography (CT) simulation imaging was performed with patients positioned using the Combi Fix system (CQ Medical, Avondale, USA). The bladder, rectum, bowel, urethra, penile bulb, pelvic bones, prostate, seminal vesicles, and external body were contoured by a clinical oncologist. The clinical target volume (CTV) included the prostate and the proximal 1 or 2 cm of seminal vesicles (SVs) exterior to the prostate depending on prostate cancer risk group. The CTV to PTV margins and offline planning dosimetric criteria are shown in Tables A1 and A2 respectively. The reference CT image was used to generate a reference plan with a 9-11 field IMRT simultaneous integrated boost technique to deliver 36.25 Gy to the prostate and proximal seminal vesicle PTV (PTV_{psv}) using the Monaco (Elekta AB, Stockholm, Sweden, V5.40.01) Treatment Planning System (TPS). All reference plans were generated with 5 segment shape optimization loops, a maximum of 60 segments, minimum segment area of 4 cm^2 and minimum of 6 monitor units per segment.

Table S1: Monaco-defined margin structures

**Name**	**Recipe**	
CTV_{psv} V_{40Gy}	(Prostate: 0cm expansion) add (Seminal Vesicles: 0cm expansion) intersect (Prostate: 1cm expansion)	
CTV_{sv}	High or upper-intermediate risk: (Prostate: 0cm expansion) add (Seminal Vesicles: 0cm expansion) intersect (Prostate: 2 cm expansion)	Lower-intermediate risk: (Prostate: 0cm expansion) add (Seminal Vesicles: 0cm expansion) intersect (Prostate: 1cm expansion)
PTV_{psv} $V_{36.25Gy}$	(CTV_{psv} V_{40Gy}: 0.5cm S/I/L/R/A, 0.3cm P expansion)	
PTV_{sv} V_{30Gy}	(CTV_{sv}: 0.5cm uniform expansion)	
PTVsv V_{30Gy}-PTV_{psv} $V_{36.25Gy}$	(CTV_{sv}: 0.5 cm uniform expansion) subtract (CTV_{psv} V_{40Gy}: 0.5 cm S/I/L/R/A, 0.3 cm P expansion)	

Table S2 on next page

Table S2: Monaco SBRT dose constraints for PACE trial

Structure	Dose Metric	Monaco tolerance
CTV_{psv} V_{40Gy}	V 40 Gy > 95 %	
PTV_{psv}	D 98 % > 34.40 Gy	-0.69 Gy
	V 36.25 Gy > 95 %	
	D 0.1 cm^3 < 48.33 Gy	
PTV_{sv} V_{30Gy} - PTV_{psv} $V_{36.25Gy}$	V 30 Gy > 95 %	
Bladder	V 18.1 Gy < 40 %	
	V 37 Gy < 5 cm^3	+ 5 cc
Bowel	V 18.1 Gy < 5 cm^3	
	V 30 Gy < 1 cm^3	
	V 18.1 Gy < 50 %	
Rectum	V 29 Gy < 20 %	
	V 36 Gy < 1 cm^3	+ 1 cm^3
FemoralHead_L/R	V 14.5 Gy < 5 %	+ 95%
PenileBulb	V 29.5 Gy < 50 %	+ 50%
Urethra	V 42 Gy < 50 %	+ 50%
Body	V 36.25 Gy < 1000 cm^3	
TissueNear	D 0.1 cm^3 < 35.16 Gy	+ 1.60 Gy
TissueDistant	D 0.1 cm^3 < 23.2 Gy	+ 2.0 Gy

Supplementary B: Contouring

Refined contours were retrospectively generated offline for both MR_{daily} and MR_{verif} images in Monaco by specially trained therapeutic radiographers on all available fractions as described below:

- MR_{daily} contouring: Bladder (see figure below), rectum, and bowel up to 3 cm cranial of the superior aspect of the prostate were recontoured in full. All other contours were generated by refining the online contours if necessary.

- MR_{verif} contouring: To initialize contouring, updated MR_{daily} contours were propagated to the MR_{verif} via (1) rigid registration for the prostate, SVs, urethra and penile bulb and (2) deformable registration for all other structures. All structures were edited as required.

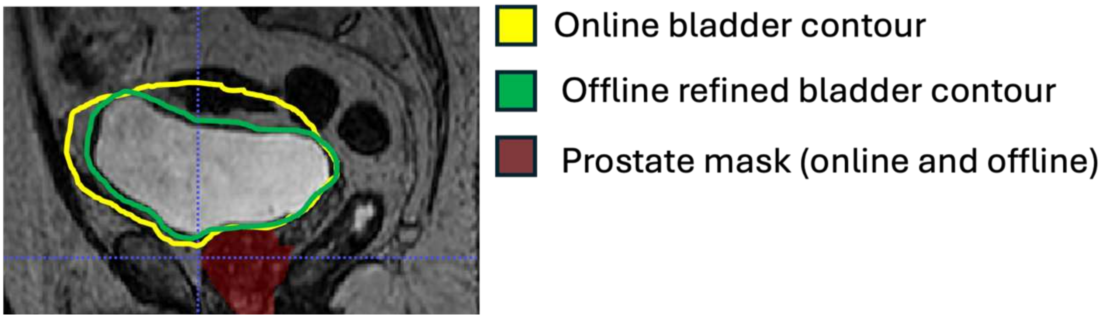

Figure S3: Example of clinical online bladder contour (yellow) used in online workflow. As the majority of the bladder is far from the prostate, it was not manually edited to conform to the bladder wall, and thus not reliable for use in calculating dose statistics in this study. This is why the online contours for each structure for each MR_{daily} image were visually inspected and edited as required offline (green contour) to ensure accurate representation of the internal anatomy.

Impact of contouring variability and contour propagation on the DPR concept:
High interobserver contour agreement on MRI for prostate RT has been previously demonstrated [12 - 14], and we don't anticipate errors greater than these published results. Even so, contouring errors and bias were mitigated in this work as follows:

A. The original online contours drawn clinically on the MR_{daily} were not contoured by the same person: a rota of clinical oncologists were responsible for defining the structures online, so there is not an inherent bias towards one specific person's contouring style.
B. All professionals responsible for contouring followed the same set of guidelines to further minimize variability and bias.

The DPR concept is less sensitive to contouring error than oART because (a) there is room for error in OAR contouring* and (b) not every contour contributes to the

discrimination between acceptable and unacceptable plans (e.g. if a PTV goal is not met, then the contours of all the other structures are irrelevant.)

Of all the structures considered in this work, DPR plans were most commonly rejected by failed PTV constraints. This means that errors/bias in prostate and SV contours would have the biggest influence in correctly classifying plans as acceptable or unacceptable, especially if these errors were to manifest as differences in target volume. However previous work has demonstrated extremely high interobserver contour agreement on prostate and SV contouring on MRL images, with no significant differences observed in target volume [14]. Although such errors are therefore presumed to be small, overestimations of target volume would erroneously suggest unacceptable target coverage (resulting in the rejection of a plan that would actually be acceptable), whereas underestimations in target volume would erroneously suggest acceptable target coverage.

Regarding backwards contour propagation: we assumed that differences in the dose distributions between a plan calculated on different MR_{daily} images over the course of the patient's 5-fraction treatment was negligible. However, we appreciate that there will be slight differences which could influence the values of the dose metrics computed in this work. This will be further investigated in future work in conjunction with the development of the software for automatically interrogating DPR plan acceptability.

**

**The 75th percentile of all OAR dose metrics over our study population (except for a single rectum constraint) was less than the corresponding clinical goal (Figure 3). This indicates that these dose metrics were not commonly violated in DPR plans and thus can still be used to discriminate between acceptable and unacceptable plans, even in the presence of possible contouring error.*

Supplementary C: Acceptability of reference plans

Table S4: Number (N) and percent of actions where the CT reference plan (CT_{ref}) was considered acceptable according to Criteria A, B, and C for each patient (n=9). *IQR* = interquartile range, *SD* = standard deviation

**Patient**	**Criteria A**		**Criteria B**		**Criteria C**		**Fractions**
	N	*%*	*N*	*%*	*N*	*%*	*N*
P1	0	0	0	0	0	0	5
P2	0	0	2	40	2	40	5
P3	0	0	0	0	0	0	5
P4	0	0	0	0	0	0	5
P5	0	0	0	0	0	0	4
P6	0	0	1	20	1	20	5
P7	0	0	0	0	2	40	5
P8	0	0	1	20	1	20	5
P9	1	20	1	20	1	20	5
**Median** [IQR]	0 [0]	**0** [0]	**0** [1]	**0** [20]	**1** [1]	**20** [20]	**5** [0]
**Mean** [SD]	0.11 [0.33]	**2** [6]	**0.56** [0.73]	**11** [15]	**0.78** [0.83]	**16** [17]	**4.89** [0.33]

Table S5: Number (N) and percent of actions where clinical reference plan ($PLAN_{ref,}$ which was the CT for fraction 1, and the online plan generated at fraction 1 for all subsequent fractions) was considered acceptable according to Criteria A, B, and C for each patient (n=9). *IQR* = interquartile range, *SD* = standard deviation

**Patient**	**Criteria A**		**Criteria B**		**Criteria C**		**Fractions**
	N	*%*	*N*	*%*	*N*	*%*	*N*
P1	1	20	1	20	1	20	5
P2	0	0	1	20	1	20	5
P3	0	0	0	0	0	0	5
P4	0	0	0	0	0	0	5
P5	0	0	0	0	0	0	4
P6	1	20	3	60	3	60	5
P7	0	0	0	40	0	40	5
P8	0	0	1	20	1	20	5
P9	1	20	1	20	1	20	5
**Median** [IQR]	0 [1]	**0** [20]	**1** [1]	**20** [20]	**1** [1]	**20** [20]	**5** [0]
**Mean** [SD]	0.33 [0.50]	**7** [10]	**1** [1]	**20** [20]	**1** [1]	**20** [20]	**4.89** [0.33]

Supplementary D: Case study of Patient 7

Patient 7 exemplifies every scenario considered and is described in detail in to aid in the interpretation of Figure 4 and the results from Table 2.

Patient 7 - Criteria A: The plan generated online at Tx2 was acceptable for all 3 subsequent fractions, as indicated by the green boxes in the 'DPR_2 ' row in Figure 4c. Although DPR_4 was also acceptable at Tx5, this plan would not hypothetically exist in the proposed workflow as DPR_2 would have been used at Tx4. These cases are color-coded in orange, and do not contribute to the results reported in Table 2.

Patient 7 - Criteria B: As with Criteria A, DPR_2 was acceptable for all subsequent fractions. Additionally, CT_{ref} (DPR_0) and MR_{ref} (DPR_1) both provided marginal target coverage at Tx5. DPR_3 and DPR_4 were both acceptable for subsequent fractions but would not have been available. Although more plans satisfied Criteria B requirements, the number of fractions where a DPR plan was acceptable (n = 3) did not increase with the loosening of constraints in this case.

Patient 7 - Criteria C: There were 2 fractions (Tx1 and Tx3) where the approved clinical plan failed to meet all target coverage criteria as estimated on MR_{verif} (e.g. 78% and 84% PTV_{psv} $V_{36.25Gy}$ [%] coverage and 74% and 84% CTV_{psv} V_{40Gy} [%] coverage for Tx1 and Tx2 respectively) due to organ motion occurring between MR_{daily} and MR_{verif} acquisitions. In both cases, DPR plans would have likely provided better target coverage than the online plan (whilst maintaining OAR sparing), which resulted in 4 fractions with an acceptable DPR plan.

In-depth analysis of Tx1:

Fraction 1 (Tx1) for patient 7 demonstrated intrafractional motion between MR_{daily} and MR_{verif} image acquisition as shown in Figure D1. This patient was treated with the ATS plan as shown. The details of clinical decisions made on this fraction were not available: the assumption from retrospective review is that as per protocol, the clinical priority of ensuring prostate coverage was achieved thus justifying the decision to proceed with treatment. It is likely that patient comfort (especially considering the full bladder) and the risk of pushing the prostate even more anteriorly (e.g. if rectal emptying was not successful upon getting the patient off the couch) contributed to this decision. Note that this was the worst case out of all fractions assessed in terms of target coverage and is not representative of general clinical practice.

The estimate of the dose-volume metrics achieved by delivering the MR_{daily}-derived plan given the MR_{verif} anatomy is shown in Table D1. Similarly, the dose-volume metrics that would have been achieved had the CT_{ref} plan been delivered are shown in Table D2. Note that although both plans are unacceptable according to Criteria A and B, the CT_{ref} plan provides better CTV and PTV coverage while simultaneously improving rectum sparing (denoted with *), making it acceptable according to Criteria C. This exemplifies how **the verification step of the oART pipeline is another potential use case for our envisioned DPR concept**: a software tool that could automatically flag up if/when DPR plans outperform online plans could be extremely beneficial in cases where large

intrafractional motion is observed, and provide an alternative to compromising coverage or getting the patient off the couch.

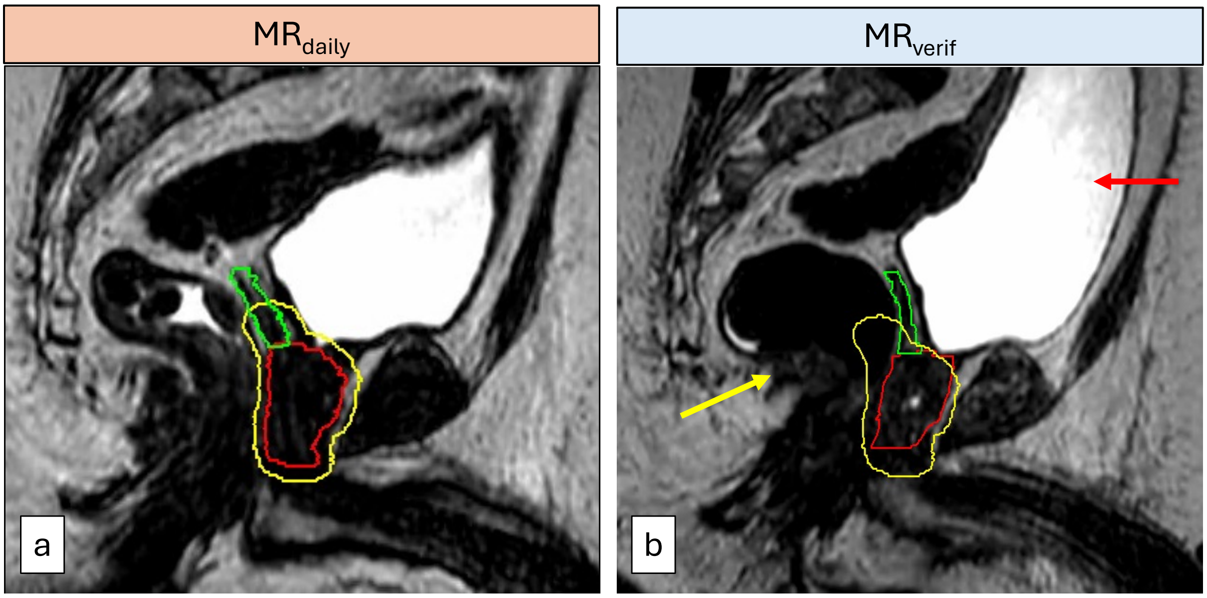

Figure S6: Intrafraction motion observed in Tx1 for Patient 7 that occurred between MR_{daily} and MR_{verif} image acquisition. The PTV generated from the MR_{daily} prostate (red) and SV (green) contours is shown as a yellow contour superimposed over both MR_{daily} and MR_{verif} images to indicate the high dose region of the clinically approved plan. (b) The increase in rectal volume (yellow arrow) contributed to an anterior rotation of the prostate and SVs resulted in reduced target coverage and increased rectal coverage. This corresponds to the dose-volume metrics for these structures being pushed out of mandatory tolerance (Table D1). Note the large increase in bladder volume (red arrow), which was assumed to contribute to the clinical decision to proceed with treatment via ATS.

Table S7: Dose statistics achieved when online plan derived from the MR_{daily} of Tx1 was recalculated onto the Tx1 MR_{verif} image for patient 7. Structures failing Criteria A and B are underlined.

Online plan Tx1 ==> MR_{verif}

Structure	Criterion	Measured value	Criteria A	Criteria B	Criteria C
$CTV_{psv}V_{40Gy}$	V40 Gy > 95%	78.43	Unacceptable	Unacceptable	-
$PTV_{psv}V_{36.25Gy}$	V36.25 Gy > 95%	74.35	Unacceptable	Unacceptable	-
$PTV_{psv}V_{36.25Gy}$	D98% > 34.4 Gy(-0.69 Gy)	26.82	Unacceptable	Unacceptable	-
Bladder	V37 Gy < 5cm^3 (+5 cm^3)	0.162	Optimal	Acceptable	-
Bladder	V18.1 Gy < 40%	2	Optimal	Acceptable	-
Rectum	V36 Gy < 1cm^3 (+1cm^3)	8.203	Unacceptable	Unacceptable	-
Rectum	V29 Gy < 20%	13.76	Optimal	Acceptable	-
Rectum	V18.1 Gy < 50%	30.57	Optimal	Acceptable	-
Bowel	V30 Gy < 1cm^3	0	Optimal	Acceptable	-
Bowel	V18.1 Gy < 5cm^3	0	Optimal	Acceptable	-

Table S8: Estimation of dose statistics that would have been achieved had the CT_{ref} plan been delivered based on the backwards propagation of MR_{verif} contours to the CT_{ref} dose distribution. Note that although the PTV clinical goals still fail (underlined text), they do so by a lesser extent than the online plan (see Table D1). Also note that all CTV and OAR clinical goals are met, including those that failed when the online plan was delivered (denoted by *).

CT_{ref} ==> Tx1

Structure	Criterion	Measured value	Criteria A	Criteria B	Criteria C
* $CTV_{psv}V_{40Gy}$	V40 Gy > 95%	95.68	Optimal	Acceptable	Acceptable
$PTV_{psv}V_{36.25Gy}$	V36.25 Gy > 95%	80.36	Unacceptable	Unacceptable	Acceptable
$PTV_{psv}V_{36.25Gy}$	D98% > 34.4 Gy(-0.69 Gy)	27.68	Unacceptable	Unacceptable	Acceptable
Bladder	V37 Gy < 5cm^3 (+5 cm^3)	2.96	Optimal	Acceptable	Acceptable
Bladder	V18.1 Gy < 40%	7.69	Optimal	Acceptable	Acceptable
*Rectum	V36 Gy < 1cm^3 (+1cm^3)	0.486	Optimal	Acceptable	Acceptable
Rectum	V29 Gy < 20%	5.01	Optimal	Acceptable	Acceptable
Rectum	V18.1 Gy < 50%	29.36	Optimal	Acceptable	Acceptable
Bowel	V30 Gy < 1cm^3	0	Optimal	Acceptable	Acceptable
Bowel	V18.1 Gy < 5cm^3	0	Optimal	Acceptable	Acceptable
